# The roles of ferroptosis regulatory gene SLC7A11 in renal cell carcinoma: A multi‐omics study

**DOI:** 10.1002/cam4.4395

**Published:** 2021-11-10

**Authors:** Fangshi Xu, Yibing Guan, Li Xue, Peng Zhang, Mingrui Li, Mei Gao, Tie Chong

**Affiliations:** ^1^ Department of Medicine Xi'an Jiaotong University Xi’an Shaanxi China; ^2^ Department of Urology The Second Affiliated Hospital of Xi'an Jiaotong University Xi'an Shaanxi Province China

**Keywords:** ferroptosis, metabolism, prognosis, renal cell carcinoma, SLC7A11, tumor immune environment

## Abstract

**Background:**

The ferroptosis inhibitory gene Solute carrier family 7 member 11 (SLC7A11) provides a new strategy for anticancer treatment. However, its function in renal cell carcinoma (RCC) remains elusive.

**Methods:**

The expression and somatic mutation information of SLC7A11 in RCC samples were determined using The Cancer Genome Atlas (TCGA), International Cancer Genome Consortium (ICGC), Gene Expression Omnibus (GEO), Oncomine, and cBioPortal databases. The prognostic value of SLC7A11 was assessed through survival analysis, Receiver operating characteristic curve (ROC) analysis, independent prognostic analysis, clinical subgroup analysis, and nomogram. Its prognostic value was also validated in the ICGC and GSE29607 cohorts. Gene set enrichment analysis (GSEA) was employed to investigate the effects of SLC7A11 on multiple metabolic pathways. The CIBERSORT algorithm and single‐sample gene set enrichment analysis (ssGSEA) method were applied to evaluate the effects of SLC7A11 on the tumor immune microenvironment (TIM). SLC7A11’s therapeutic correlations were analyzed using the GSE87121, GSE67501, and GSDC datasets. Finally, the biofunctions of SLC7A11 in renal cancer cells and ferroptosis were ascertained by MTT, wound healing, transwell, and western blot assays.

**Results:**

Through multiple datasets, SLC7A11 was found to be markedly upregulated in RCC. In terms of prognosis, SLC7A11 overexpression conferred a worse prognosis and was identified as an independent prognostic factor. Its prognostic value was validated in ICGC cohort. Moreover, high SL7CA11 expression could stimulate nucleotides, fatty acids, and amino acid metabolism to meet the proliferative consumption of tumor cells. As for the immune effect, SLC7A11 suppressed antitumor immunity by reducing the abundances of CD8+ T and NK cells. Regarding the therapeutic response, SLC7A11 expression was not correlated with the sensitivities of most chemotherapy and targeted drugs. Finally, SLC7A11 promoted the proliferation, migration, and invasion of renal cancer cells by enhancing GPX4 output, which in turn inhibits ferroptosis.

**Conclusions:**

SLC7A11 not only deeply influences RCC prognosis and TIM, but also promotes RCC progression by inhibiting ferroptosis and inducing metabolic reprogramming. In addition, SLC7A11 weakly affects the therapeutic effect and sensitivities of multiple chemotherapy and targeted drugs.

## Graphical abstract

We comprehensively probed into the roles of Solute carrier family 7 member 11 (SLC7A11), a ferroptosis regulatory gene in prognosis, immune microenvironment, biological metabolism, therapeutic correlation, and pro‐oncogenic abilities of renal cell carcinoma (RCC) through bioinformatic analyses and experimental validation.

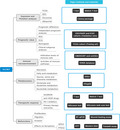

## 1 INTRODUCTION

Renal cell carcinoma (RCC) is a prevalent type of urologic cancer with an incidence of 12.4 per 100,000.^1^ In 2021, approximately 76,000 new cases and approximately 13,000 deaths were estimated from the disease in the United States.^2^ Despite significant improvements in RCC diagnosis and treatment, the incidence and death of RCC continue to increase annually.^1^, ^3^ Either partial or radical nephrectomy is the mainstay of therapeutic approaches; the 5‐year overall survival rate (OSR) of postoperative patients is as high as 81%.^4^ However, over 25% of RCC patients present with metastatic signs at the time of diagnosis, with a 1‐year OSR of 29%.^5^ With the successful application of immune checkpoint inhibitors (ICIs), tyrosine kinase inhibitors (TKIs), and vascular endothelial growth factor (VEGF)‐targeted drugs, there is an increased demand for individualized and integrated treatments for RCC, especially for metastatic RCC.^6^ Therefore, exploring the molecular mechanism of RCC onset and progression, searching for new targets, and developing accurate prognostic systems are extremely meaningful.

Ferroptosis is a new form of programmed cell death, which was first coined in 2013 and provides a promising anticancer strategy.^7^ It is well‐known that the dysfunction of the cell antioxidant GPX4 exerts a promoting effect on the ferroptosis process. GPX4 synthesis relies on intracellular cystine, which is transported by the xCT system.^8^ Solute carrier family 7 member 11 (SLC7A11) constitutes the major component of the xCT system and is capable of inhibiting ferroptosis by promoting cystine uptake.^8^ Accumulating evidence has shown that SLC7A11 is overexpressed in multiple cancers and can promote tumor progression by retarding ferroptosis, leading to metabolic reprogramming.^9^ Therefore, SLC7A11 shows great potential as a new target for cancer therapy.^10^ However, the biofunctions of SLC7A11 in RCC have not been investigated.

In view of this, the original intention of our study was to comprehensively reveal the functions of SLC7A11 in RCC from six perspectives. It included its expression and somatic mutation information, prognostic value, impact on tumor immune microenvironment (TIM), effects on biosynthetic metabolism and ferroptosis process, and therapeutic correlation. Moreover, through in vitro experiments, we confirmed its pro‐oncogenic effects on the proliferation, migration, and invasion of renal cancer cells. These findings will provide new insights into the treatment, clinical assessment, and molecular mechanisms of RCC.

## 2 MATERIALS AND METHODS

### 2.1 Data source

Clinical information and transcriptome data were obtained from The Cancer Genome Atlas (TCGA) (https://portal.gdc.cancer.gov/), International Cancer Genome Consortium (ICGC) (https://dcc.icgc.org/releases), and Gene Expression Omnibus (GEO) (https://www.ncbi.nlm.nih.gov/geo/) public databases. In the TCGA database, the data and workflow types of transcriptome profiling were “Gene Expression Quantification” and “HTSeq—FPKM,” respectively. The data format of the clinical information was “bcr xml.” The project was “TCGA‐KIRC,” which provided 537 RCC and 72 normal samples. In the ICGC database, we chose “RECA‐EU” project for bioinformatic analyses. Gene expression data and clinical information were extracted using Perl language (Version 5.30.2). In the GEO database, GSE53757, GSE66271, GSE168845, GSE15641, GSE105261, GSE150404, GSE53757, GSE87121, GSE67501, and GSE29609 were used for gene expression, prognostic, and therapeutic response analyses. The description, specified uses, and sample information of the GEO datasets are presented in Table 1. The extraction of gene expression data and clinical information was also achieved using the Perl language (version 5.30.2). To ensure comparability between different datasets, gene expression data were standardized by log_2_ transformation. Specifically, transcriptome data of GSE29609 were standardized using the base 10 logarithm, according to the data descriptions.

**TABLE 1:** The description and purpose of used datasets in the present study

Dataset	Available sample size T/N	PMID	Description	Purpose
TCGA	537/72	—	KIRC project	1. Expression analysis 2. Training cohort of prognostic value
ICGC	91/0	—	RECA‐EU project	1. Expression analysis 2. Validation cohort of prognostic value
GSE29609	39/0	22626276	Whole genome expression and clinical characteristics of ccRCC patients	Validation cohort of prognostic value
GSE53757	73/73	24962026	Gene expression profile of RCC and matched normal renal tissues	Expression analysis
GSE66271	13/13	26859141	Gene expression profile of mRCC and matched normal renal tissues	Expression analysis
GSE168845	4/4	NA	Gene expression profile of RCC (stages III–IV) and matched normal renal tissues	Expression analysis
GSE15641	45/23	16115910	Transcriptional profiling of ccRCC, pRCC, chrRCC, RO, and normal samples	Expression analysis
GSE105261	35/9	30131446	Transcriptional profiling of primary RCC, mRCC, and normal samples	Expression analysis
GSE150404	60/0	NA	Expression data from RCC patients with different stages	Expression analysis
GSE87121	10/0	NA	Expression data in RCC samples from patients who presented sorafenib resistance or effective	Therapeutic correlation
GSE67501	11/0	27491898	Expression data in RCC samples from patients who did or did not respond to nivolumab	Therapeutic correlation

Abbreviations: ccRCC, clear cell renal cell carcinoma; chrRCC, chromophobe RCC; EU, Europe; KIRC, kidney renal clear cell carcinoma; mRCC, metastatic RCC; NA, not available; pRCC, papillary RCC; RECA, renal cell cancer; RO, renal oncocytoma; T/N, Tumor samples versus normal samples.

### 2.2 Expression and mutation analyses

The differences in SLC7A11 expression between normal and RCC samples were compared using either the *t*‐test or the Welch’s *t*‐test. Moreover, the relationships between SLC7A11 expression and RCC clinicopathological features were determined using either the Kruskal–Wallis test or one‐way analysis of variance. The Oncomine database (https://www.oncomine.org/) was employed to analyze the pan‐cancer expression of SLC7A11 and conduct a meta‐analysis of SLC7A11 based on three renal cancer datasets. The analytical thresholds were as follows: *p*‑value = 0.05, fold change = 2, gene rank was “Top 10%,” and data type was “mRNA.”

The cBioPortal database (http://cbioportal.org) can provide somatic mutation information for SLC7A11.^11^ “OncoPrint tab” exhibited the SLC7A11 mutant samples across four RCC studies (a total of 662 samples) and displayed the mutation type of SLC7A11.

### 2.3 Prognostic analyses

The prognostic value of SLC7A11 was evaluated in six ways in the TCGA cohort. (i) According to the cutoff value of SLC7A11 expression, 537 RCC samples were divided into high‐ and low‐SLC7A11 groups. The survival difference between the different SLC7A11 expression groups was compared using the Kaplan–Meier method. The optimal cutoff value was determined using the Cutoff Finder online tool (http://molpath.charite.de/cutoff).^12^ (ii) Receiver operating characteristic curve (ROC) was used to assess the predictive accuracy of SLC7A11 and other clinical parameters. (iii) Decision curve analysis (DCA) was used to determine whether introducing SLC7A11 expression into the traditional prognostic model could increase the clinical net benefit. Based on the multivariate logistic regression algorithm, the traditional prognostic model consisted of age, histological grade, and TNM staging. (iv) Cox univariate and multivariate analyses were successively conducted to identify the independent prognostic factors of RCC. (v) Clinical subgroup analyses were performed to estimate the applicable range of SLC7A11 in the RCC prognosis analysis. (vi) Based on the multivariate logistic regression algorithm, we constructed a nomogram combining RCC clinicopathological features (age and TNM staging) and SLC7A11 expression levels to predict the OSR of individuals at 1, 3, and 5 years. A calibration plot was constructed to test its predictive accuracy.

The prognostic value of SLC7A11 was also validated in the ICGC and GSE29607 cohorts. Survival difference analysis, independent prognostic analysis, and ROC test were performed in two validation cohorts. The optimal cutoff value for SLC7A11 expression was used as a grouping criterion. Heatmaps containing the distributions of SLC7A11 expression and clinical characteristics of RCC were produced using R software (Version 3.6.3).

### 2.4 Metabolomic analyses

GSEA^13^ was used to investigate the effects of SLC7A11 on some important metabolic pathways. “Phenotype labels” were set as high expression SLC7A11 samples versus low expression ones. The number of permutations was set to 1000 for the GSEA operations. There is no collapse of gene symbols. The gene sets required for GSEA were selected from MSigDB (Molecular Signatures Database).^14^ We assessed the influence of SLC7A11 on glycolysis, fatty acids, nucleotides, and amino acid metabolism from a metabolic perspective. On the other hand, considering the crucial role of SLC7A11 in ferroptosis regulation, we also analyzed its impact on ferroptosis‐related process. A total of 12 gene sets were applied for GSEA, including “GO Glycolytic Process,” “Hallmark Glycolysis,” “Reactome Glycolysis,” “Module 306,” “Ferroptosis,” “Hallmark Oxidative Phosphorylation,” “Hallmark Reactive Oxygen Species Pathway,” “GOBP Iron ion Transport,” “KEGG Glycine Serine and Threonine Metabolism,” “Reactome Glutamate and Glutamine Metabolism,” “Module 337,” and “Hallmark Fatty acid Metabolism.” Descriptions of these gene sets are shown in Table S1.

### 2.5 Immune analyses

The CIBERSORT algorithm is a computational method for quantifying cell fractions based on gene expression profiles (GEPs) of tissues.^15^ Using this algorithm, the immune abundances of 22 lymphocyte subtypes in each RCC sample were calculated. Then, we compared the differences in infiltration levels of immune cells between high and low expression SLC7A11 groups via the “Limma” package in R software. The activities of 13 immune‐related pathways were quantified using ssGSEA (single‐sample gene set enrichment analysis) method, which was implemented by the “GSVA” package.^16^ Moreover, we explored the associations between the enrichment levels of antitumoral immune effector cells and SLC7A11 expression using the Spearman correlation test. The TIMER web server is a comprehensive resource for systematic analysis of immune infiltrates across diverse cancer types (https://cistrome.shinyapps.io/timer/).^17^ The somatic copy number alteration (SCNA) module was used to estimate the influence of SLC7A11 SCNA on the abundance of immune infiltration.

### 2.6 Therapeutic correlation analyses

In the current study, we also investigated the relationship between SLC7A11 expression and the efficacy of multiple drugs from five perspectives. First, we compared the expressive difference in SLC7A11 among different clinical response categories in the TCGA cohort using the Wilcoxon test, including progressive disease (PD), stable disease (SD), complete response (CR), and partial response (PR). Second, using the GSE87121 dataset, the expressive difference in SLC7A11 between sorafenib‐response and sorafenib‐nonresponse (a multikinase inhibitor) patient was determined by the *t*‐test. The effect of SLC7A11 expression on the therapeutic response rate of sorafenib was ascertained by the Wilcoxon test. Third, using the GSE67501 dataset, we explored the relationships between the therapeutic response of nivolumab (a PD‐1 inhibitor) and SLC7A11 expression using the same method as sorafenib. Fourth, given that patients with a high expression of ICs could benefit from ICI therapy,^18^ we probed the expressive correlations between SLC7A11 and six important ICs (PD‐1, CTLA4, LAG3, HAVCR2, TIGIT, and BTLA) based on the Spearman test. Fifth, using the Genomics of Drug Sensitivity in Cancer database (www.cancerRxgene.org),^19^ we investigated the correlation between SLC7A11 and susceptibility to 266 drugs.

### 2.7 Cell culture and transfection

The normal kidney tubular epithelial cell line HK‐2 and renal cancer cell lines 786‐O and A498 were purchased from Procell Life Science and Technology Company. HK‐2 cells were cultured in minimum essential medium containing 10% fetal bovine serum (FBS) and 1% penicillin/streptomycin. 786‐O and A498 cells were cultured in RPMI‐1640 medium (Roswell Park Memorial Institute [RPMI]) containing 10% FBS and 1% P/S. The culture conditions were 37°C, 5% CO^2^, and 95% humidity.

Small interfering RNA (siRNA) for SLC7A11 silencing was designed and synthesized by GenePharma Biotechnology. pcDNA3.1 plasmid for SLC7A11 overexpression was purchased from Fenghui Biotechnology Company. Lipofectamine™ 2000 was used for transfection (Thermo Fisher Scientific).

### 2.8 Real‐time quantitative PCR (RT‐qPCR)

Total RNA was extracted using TRIzol reagent (Thermo Fisher Scientific). Reverse transcription (RT) was performed using the PrimeScript™ RT reagent Kit with gDNA Eraser (Takara). Transcript levels were measured using SYBR‐Green PCR Reagent (Takara). RT‐qPCR was performed using the ABI Prism 7900 sequence detection system. GAPDH was used as an internal control to normalize PCR data. SLC7A11 expression was calculated based on the 2^−ΔΔCT^ method. The primer list is shown in Table S2.

### 2.9 MTT assay

After siRNA and pc‐SLC7A11 transfection, renal cancer cells were seeded into 96‐well plates at a concentration of 5 × 10^4^ cells per well and incubated for 24, 48, 72, and 96 h. For detection, MTT reagent (Solarbio) was added to each well and the cells were incubated at 37°C for 4 h. After removing the medium, 150 μl of DMSO was added and agitated for 15 min to dissolve the formazan crystals. The optical density (OD) was measured using a microplate reader at 490 nm wavelength.

### 2.10 Cell cycle analysis

When transfected cells reached the logarithmic phase, they were trypsinized and centrifuged at 503 *g*  for 15 min. After removing the supernatant, the cells were washed twice with phosphate buffer solution (PBS) and resuspended in PBS 0.5 ml. The cells were then placed in pre‐cooled 70% anhydrous ethanol at −20℃ for 1 h. Fixed cells were harvested by centrifugation and washed twice with PBS. Next, the cells were resuspended in PBS 0.5 ml and 100 µl RNase A (50 µl/ml) was added for digestion at 37°C for 30 min. PI dye solution (100 µl) was added for staining at 4°C in the dark for 30 min. Flow cytometric analysis was performed using the Becton Dickinson FACScan system.

### 2.11 Wound healing assay

Renal cancer cells transfected with si‐SLC7A11 or pc‐SLC7A11 were seeded into a 6‐well plate (5 × 10^4^ cells per well). When the cell confluence reached above 90%, a linear wound was created using a sterile pipette tip. Dropped cells were discarded by washing twice with PBS. Cell migration was monitored by a microscope after 24 h of incubation. The migratory abilities of the cells were quantified by the wound width rate. Wound width rate = (scratch width at 0 h *minus* that at 24 h *divided by* initial width)  × 100%.

### 2.12 Transwell assay

The invasive abilities of the cells were estimated using 24‐well transwell chambers (Corning). Transfected cells (5 × 10^4^ cells per well) were seeded into the upper chamber with serum‐free medium. The top surface of the upper chamber was coated with Matrigel. RPMI‐1640 medium supplemented with 10% FBS was added to the lower chamber. After 24 h of incubation, the medium was discarded, and noninvasive cells were removed by washing twice with PBS and wiped with a cotton swab. The invasive cells were fixed with 4% paraformaldehyde for 15 min and stained with 0.1% crystal violet for 5 min at room temperature. The stained cells were counted at 100‐fold magnification per three random fields of view under a microscope.

### 2.13 Western blot

After trypsin digestion, PBS washing, and centrifugation, total proteins from transfected cells were extracted using radioimmunoprecipitation assay buffer reagent (Solarbio). A BCA Protein Quantification Kit (Vazyme) was used to measure the total protein concentration of the clarified lysate. Cell lysates (20 μl) were loaded in each lane of 10% SDS‐PAGE gels and the protein samples were separated by constant voltage electrophoresis for 2.5 h (110 V). The separated proteins were transferred onto PVDF membranes by 1.5 h electrophoresis (Millipore). The membranes were washed in TBST and blocked with 5% fat‐free milk powder in TBST. Primary antibodies were added, and the membranes were incubated overnight at 4°C. After washing thrice with TBST (Tris‐buffered saline with Tween), the membranes were incubated with secondary antibodies at room temperature for 2 h. OD values of the targeted bands were analyzed using Gel‐Pro Analyzer software. Recombinant rabbit monoclonal antibodies against target genes were used as primary antibodies (Thermo Fisher). Goat anti‐rabbit IgG (H+L) secondary antibody was used as the secondary antibody (Thermo Fisher).

### 2.14 Statistical analysis

All statistical analyses were performed using R software (Version 3.6.2) and GraphPad Prism (Version 8.01). All data visualization was fulfilled using R software (Version 3.6.2). Statistical significance was set at *p* < 0.05.

## 3 RESULTS

**FIGURE 1:**
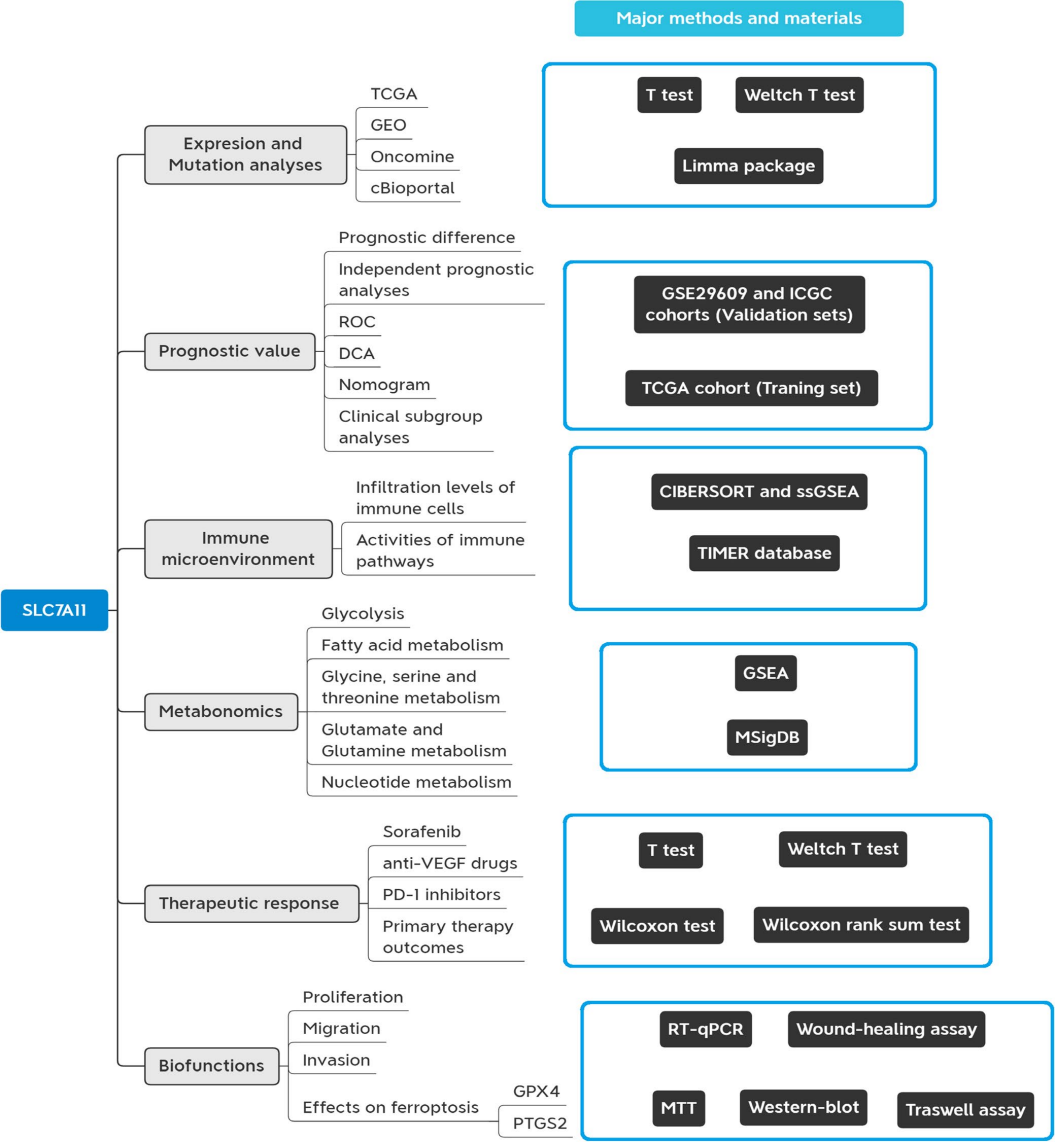
Flow chart of present study. DCA, decision curve analysis; ROC, receiver operating characteristic curve

A flow chart is shown in Figure 1. Using multi‐omics bioinformatics approaches, we probed the roles of SLC7A11 in expression, mutation, prognosis, TIM, metabolism, treatment, and biofunctions in RCC. Its prognostic value was also validated in the ICGC and GSE29609 cohorts. We also ascertained that SLC7A11 has an RCC‐promoting ability at the cellular level. The clinical characteristics of the TCGA, ICGC, and GSE29606 cohorts are shown in Table S3–S5.

### 3.1 SLC7A11 is significantly upregulated in RCC

**FIGURE 2:**
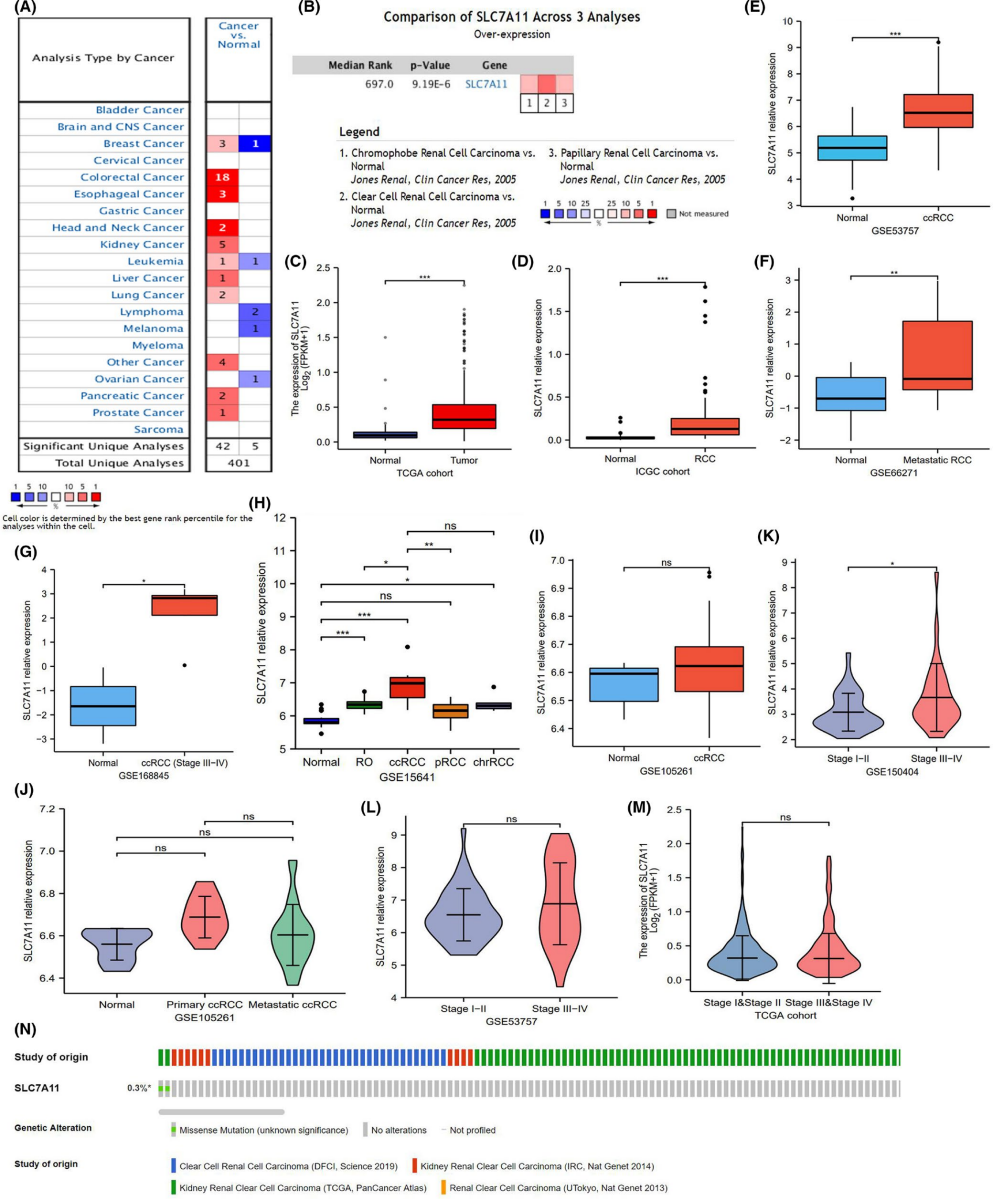
SLC7A11 expression in RCC samples. (A) Pan‐cancer analysis of SLC7A11 in Oncomine database. (B) Meta‐analysis of SLC7A11 based on three renal cancer datasets in Oncomine database. (C) SLC7A11 expression in TCGA dataset. (D) SLC7A11 expression in ICGC dataset. (E–M) SLC7A11 expression in multiple GEO datasets. (N) The somatic mutation information of SLC7A11 in cBioPortal database. chrRCC, chromophobe renal cell carcinoma; pRCC, papillary renal cell carcinoma; RCC, renal cell carcinoma; RO, renal oncocytoma; *p < 0.05, **p < 0.01, and ***p < 0.001

Oncomine pan‐cancer analysis showed that SLC7A11 overexpression was observed in multiple cancers (Figure 2A). A meta‐analysis of three RCC datasets from Jones et al.^20^ revealed that SLC7A11 was upregulated in three subtypes of renal carcinoma. Meanwhile, SLC7A11 expression displayed the same ascending trend in TCGA, ICGC, and GEO datasets (Figure 2C–G). Similar to the results of the Oncomine meta‐analysis (Figure 2A), SLC7A11 was upregulated in ccRCC, chrRCC (chromophobe renal cell carcinoma), and renal oncocytoma in the GSE15641 dataset (Figure 2H). However, there was no significant difference in SLC7A11 expression between normal and ccRCC samples in the GSE105261 dataset (Figure 2I,J). Interestingly, the expression of SLC7A11 in clinical stages III/IV was not always higher than that in clinical stages I/II (Figure 2K–M).

In addition, the somatic mutation of SLC7A11 was barely visible in RCC samples (*n* = 2/667, 0.3%), which suggested that the aberrant expression of SLC7A11 may result from transcriptional or posttranslational regulation (Figure 2N). Collectively, SLC7A11 upregulation was not only confirmed in multiple datasets, but also in multiple pathological subtypes of renal carcinoma.

### 3.2 SLC7A11 provides valuable information for RCC prognostic assessment

**FIGURE 3:**
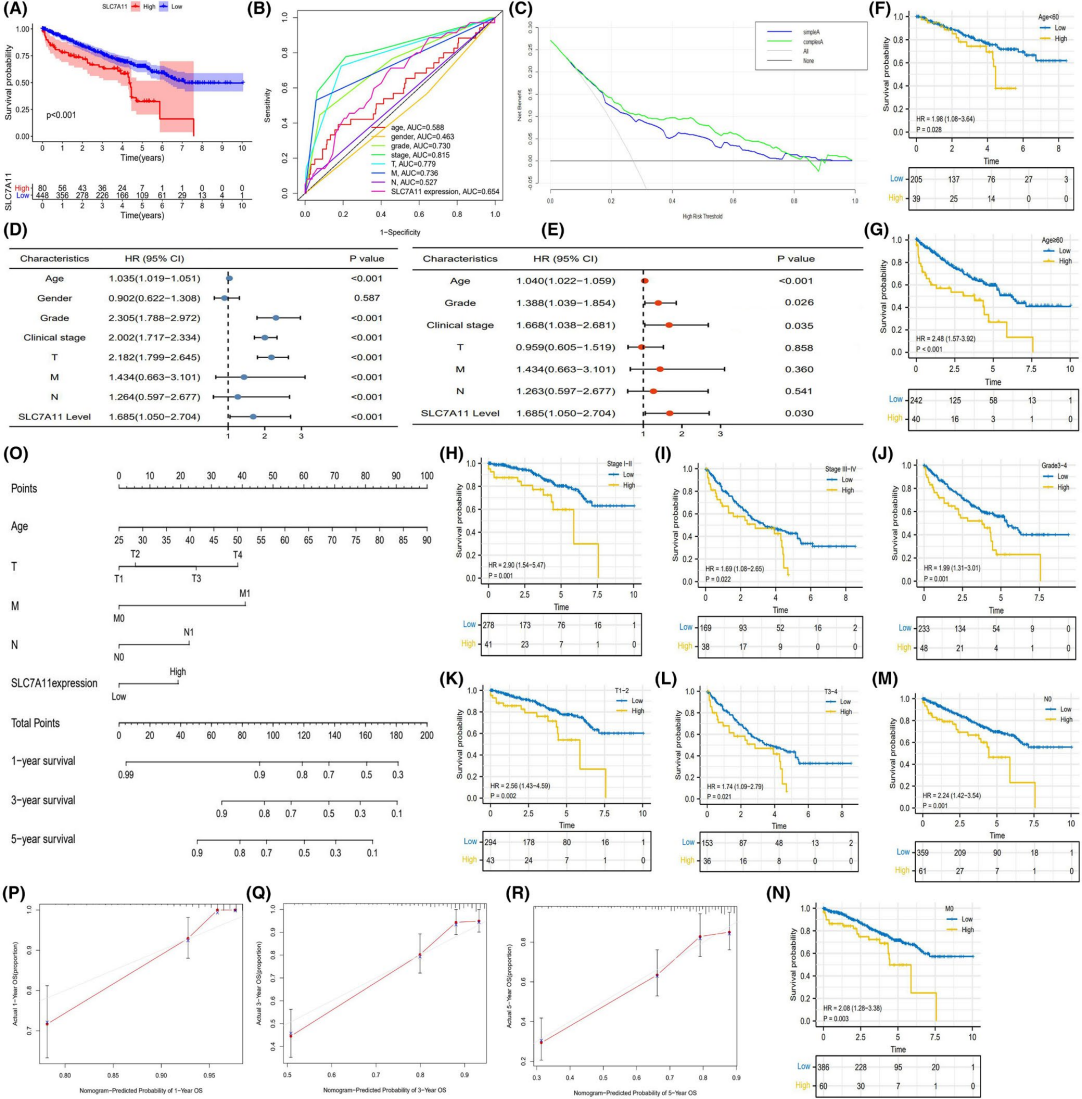
The prognostic value of SLC7A11 in TCGA cohort. (A) The survival difference between high and low SLC7A11 expressions. (B) ROC in TCGA cohort. (C) DCA curve of SLC7A11. Different curves represent two kinds of RCC prognostic models (based on multivariate logistic regression analysis). “Sample A” represents the prognostic model consisting of age, histological grade, and TNM staging. “Complex A” represents the improved prognostic model consisting of age, histological grade, TNM staging, and SLC7A11 expression. (D) Cox univariate regression analysis in TCGA cohort. (E) Cox multivariate regression analysis in TCGA cohort. (F–N) Clinical subgroup analyses. (O) The nomogram consisting of age, TNM staging, and SLC7A11 expression. (P–R) Calibration plots of 1‐, 3‐, and 5‐year OSR. DCA, decision curve analysis; OSR, overall survival rate; RCC, renal cell carcinoma; ROC, receiver operating characteristic curve

We further investigated the prognostic value of SLC7A11 in the TCGA cohort. According to the optimal cutoff value of SLC7A11 relative expression (0.6013), 528 RCC samples were divided into high and low SLC7A11 expression groups (Figure S1). High SLC7A11 expression conferred a poor prognosis, leading to a 3‐year survival rate of 62.9%, whereas that of low SLC7A11 expression was 76.2% (Figure 3A). Although SLC7A11 had good sensitivity and specificity in predicting survival status (AUC = 0.654), it did not reveal a preponderance over histological grade, clinical stage, and TM stages (Figure 3B). DCA analysis indicated that the clinical benefit could be increased after adding SLC7A11 expression to the traditional prognostic model (age, histological grade, and TNM staging) (Figure 3C). Univariate and multivariate regression analyses identified only age, histological grade, and clinical stage, and SLC7A11 expression (HR = 1.685, *p* = 0.030) was identified as an independent prognostic factor of RCC (Figure 3D,E).

Through clinical subgroup analyses, SLC7A11 could distinguish survival differences of patients in all age groups, clinical stages I–IV, grades 3–4, T1–4, N0, and M0 stages (Figure 3F–N), which indicated that SLC7A11 had good applicability in RCC prognostic analysis. In order to predict the OSR of individual at 1, 3, and 5 years, we constructed a nomogram consisting of age, TNM staging, and SLC7A11 expression (Figure 3O). Calibration plots showed that the predicted OSR well matched the real survival rate (Figure 3P–R). Based on these findings, it is conceivable that SLC7A11 contributes to the prognostic assessment of RCC patients.

### 3.3 The prognostic value of SLC7A11 also can be validated in the ICGC cohort

**FIGURE 4:**
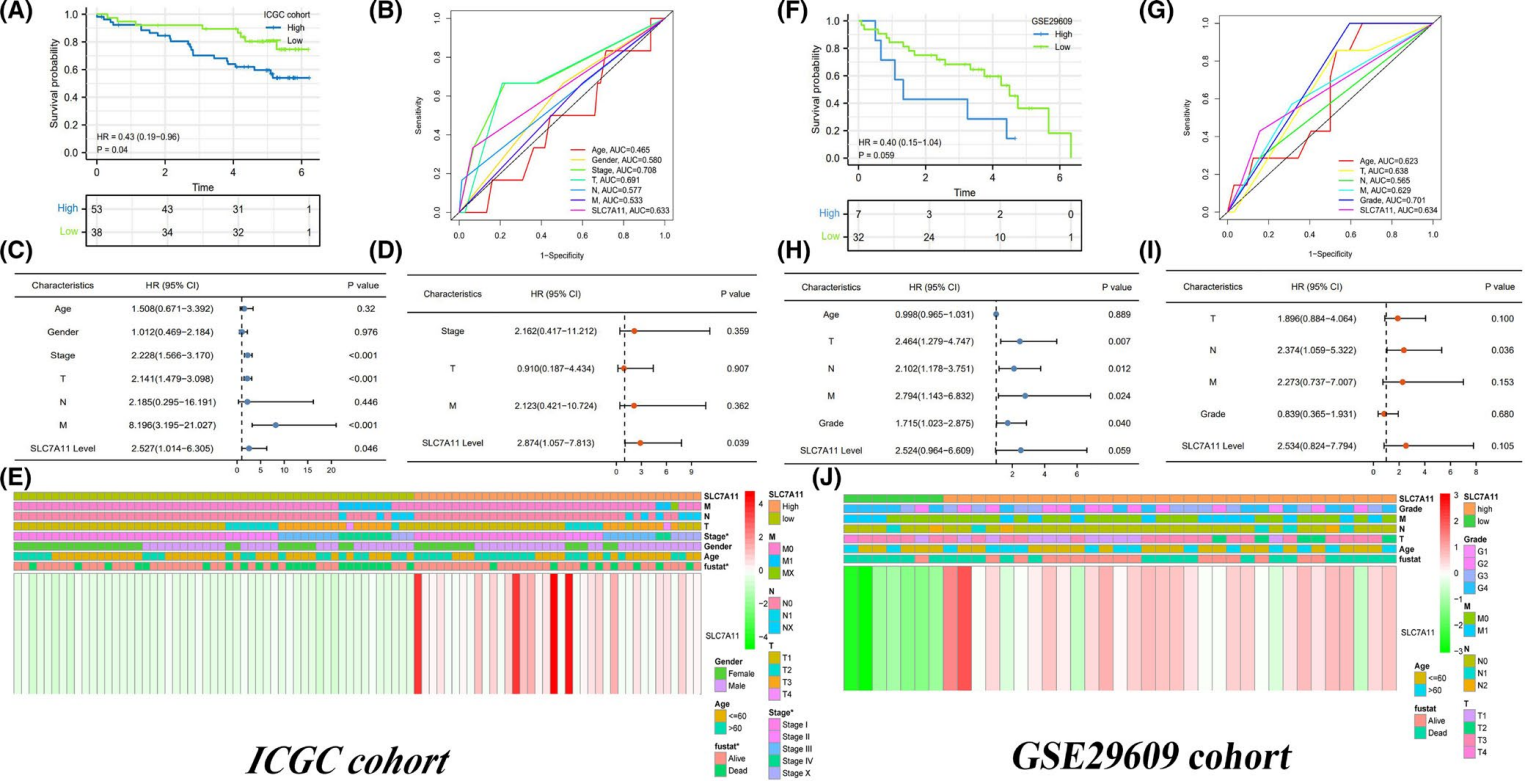
The prognostic value of SLC7A11 in validation cohorts. (A) Survival difference in ICGC cohort. (B) ROC in ICGC cohort. (C) Cox univariate regression analysis in ICGC cohort. (D) Cox multivariate regression analysis in ICGC cohort. (E) SLC7A11 heatmap in ICGC cohort. (F) Survival difference in GSE29609 cohort. (G) ROC in GSE29609 cohort. (H) Cox univariate regression analysis in GSE29609 cohort. (I) Cox multivariate regression analysis in GSE29609 cohort. (J) SLC7A11 heatmap in GSE29609 cohort. CI, confidence Interval; HR, hazard ratio; *p < 0.05

In addition, we tested the prognostic value of SLC7A11 in the ICGC and GSE29609 cohorts. As previously found in the TCGA cohort, high expression of SLC7A11 resulted in a poor survival outcome (Figure 4A). SLC7A11 presented moderate predictive performance for living states (AUC = 0.633, Figure 4B). In addition, univariate and multivariate analyses revealed that SLC7A11 expression level was the only independent prognostic indicator of RCC (Figure 4C,D).

However, a different picture emerged in the GSE29609 cohort. Although the OSR of RCC patients with high SLC7A11 expression was intuitively lower than that of patients with low expression, it was not statistically significant (*p* = 0.059) (Figure 4F). SLC7A11 predictive ability in the GSE29609 cohort was similar to that in the ICGC cohort (AUC = 0.634, Figure 4G). Unfortunately, SLC7A11 was not identified as an independent prognostic factor in the GSE29609 cohort, and only the N stage played this role (Figure 4H,I). The heatmaps of SLC7A11 in the two validation cohorts are shown in Figure 4E,J.

### 3.4 SLC7A11 inhibits ferroptosis and confers metabolic reprogramming

**FIGURE 5:**
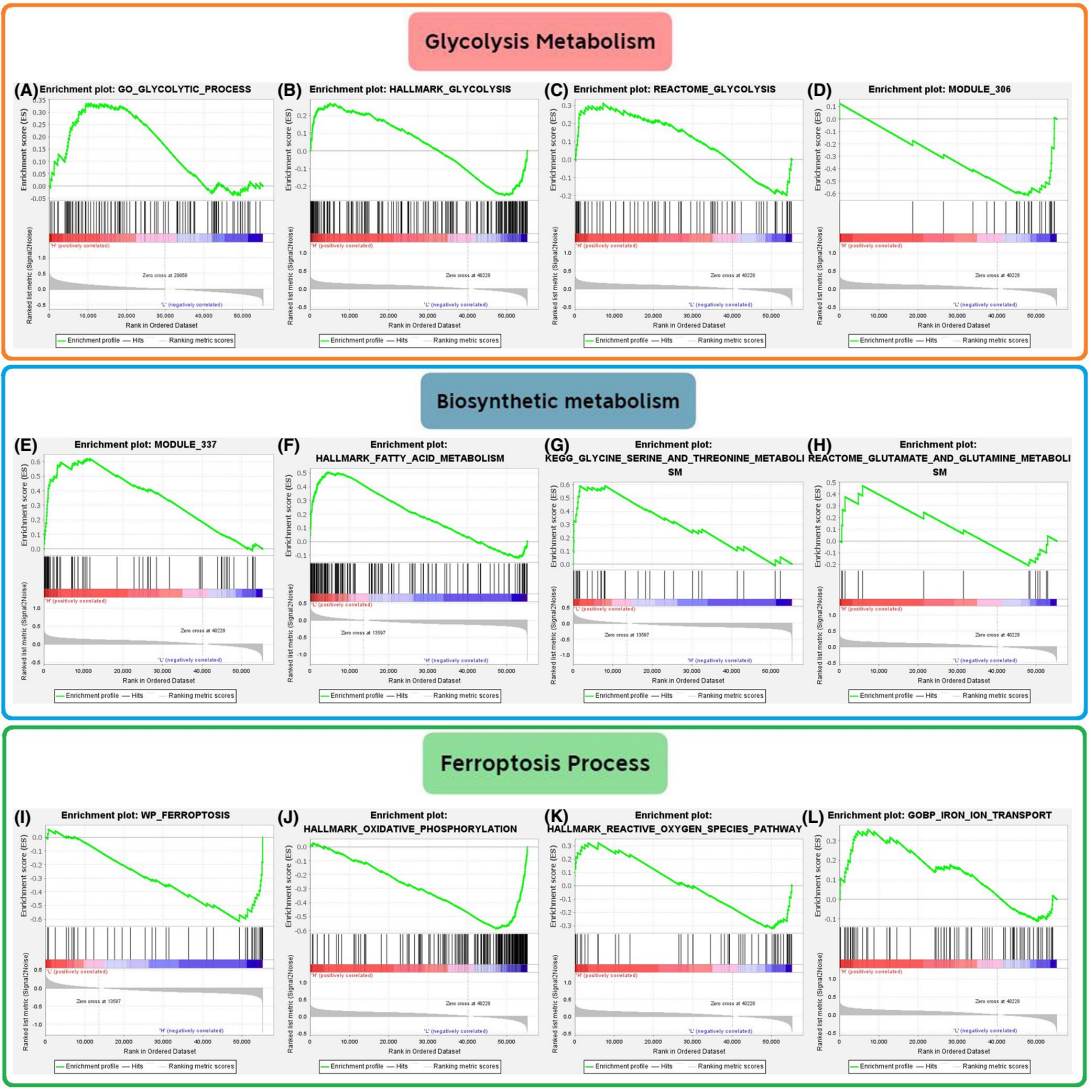
The effects of SLC7A11 on biosynthetic metabolism and ferroptosis based on GSEA. (A–D) The effects of SLC7A11 on glycolysis‐related gene sets. (E–H) The effects of SLC7A11 on “nucleotide,” “fatty acid,” “glutamate and glutamine,” and “glycine serine and threonine” metabolic gene sets. (I–L) The effects of SLC7A11 on ferroptosis‐related gene sets. Module 306 gene set represents “Glycolysis and TCA cycle”; Module 337 gene set represents “Nucleotide metabolism.” GSEA, gene set enrichment analysis

Metabolic reprogramming is a vital hallmark of cancer and plays a critical role in tumor onset and progression (Table 2). Glycolysis, also known as the Warburg effect, can fulfill the biosynthetic requirements of tumor cell proliferation, such as nucleotides and NADPH.^21^ However, none of the glycolysis‐related sets were enriched in the high SLC7A11 expression group (Figure 5A–D and Table 3). For other nutrients, nucleotides, fatty acids, glutamine, glycine, serine, and threonine metabolism all supply the synthetic material and energy source needed for tumor cell proliferation.^22^, ^23^, ^24^, ^25^ Therefore, “nucleotide metabolism” (*p* = 0.009), “fatty acid metabolism” (*p* = 0.049), and “glycine, serine, and threonine metabolism” (*p* = 0.036) were enriched in the high SLC7A11 expression group, but not for “glutamate and glutamine metabolism” (*p* = 0.277) (Figure 5E–H).

**TABLE 2:** Functions of biological metabolism in cancer progression

Name	PMID	Function in cancer	Enrichment phenotype
Glycolysis	31629815 30822194	1. Fulfill the biosynthetic requirements of tumor cell proliferation. 2. Enhance cell resistance to antitumor therapies through accumulation of lactate.	NS
Nucleotide metabolism	24486217	1. Provide a high level of dNTPs pool for DNA replication and repair. 2. Increase genomic instability to induce cancer initiation.	High
Fatty acid metabolism	31819192	1. Provide structural components of the membrane matrix. 2. Serve as secondary messengers and fuel sources. 3. Promote cancer progression through remodeling TIM.	High
Glutamate and glutamine metabolism	23999442	Involve in energy formation, redox homeostasis, macromolecular synthesis, and signaling in cancer cells.	NS
Glycine serine and threonine metabolism	24657017	Provide the essential precursors for the synthesis of proteins, nucleic acids, and lipids.	High

Abbreviations: NS, Not significant; TIM, tumor immune microenvironment. High, high SLC7A11 expression group.

**TABLE 3:** Metabolic reprogramming caused by SLC7A11

Gene set	Enrichment score (ES)	Normalized (NES)	Nominal *p* value
Glycolysis metabolism			
GO glycolytic process	0.294	1.063	0.377
Hallmark glycolysis	0.267	0.946	0.507
Glycolysis (module 306)	−0.615	−1.486	0.142
Reactome glycolysis	0.311	0.961	0.477
Biosynthetic metabolisms			
Nucleotide metabolism (module 337)	0.619	1.952	0.009
Hallmark fatty acid metabolism	0.505	1.657	0.049
Reactome glutamate and glutamine metabolism	0.469	1.206	0.277
KEGG glycine serine and threonine metabolism	0.591	1.739	0.036
Ferroptosis process			
WP ferroptosis	−0.617	−1.846	0.012
Hallmark oxidative phosphorylation	−0.586	−1.538	0.135
Hallmark reactive oxygen species pathway	−0.324	−0.956	0.522
GOBP iron ion transport	0.357	1.293	0.202

Abbreviations: BP, biological process; GO, Gene Ontology; GSEA, Gene set enrichment analysis; KEGG, Kyoto Encyclopedia of Genes and Genomes; WP, Wikipedia.

SLC7A11 can promote GPX4 synthesis by increasing cystine uptake, which in turn suppresses ferroptosis. As expected, “WP Ferroptosis” was found to be enriched in the low SLC7A11 expression group (*p* = 0.012) (Figure 5I). Intriguingly, “oxidative phosphorylation” (*p* = 0.135) and “reactive oxygen species pathway” (*p* = 0.522), which are effective links in ferroptosis, did not show significant enrichment differences between high and low SLC7A11 expression groups (Figure 5J,K). SLC7A11 expression levels did not alter the enrichment of iron ion transport (*p* = 0.202) (Figure 5L).

### 3.5 High SLC7A11 expression retards antitumor immune process

**FIGURE 6:**
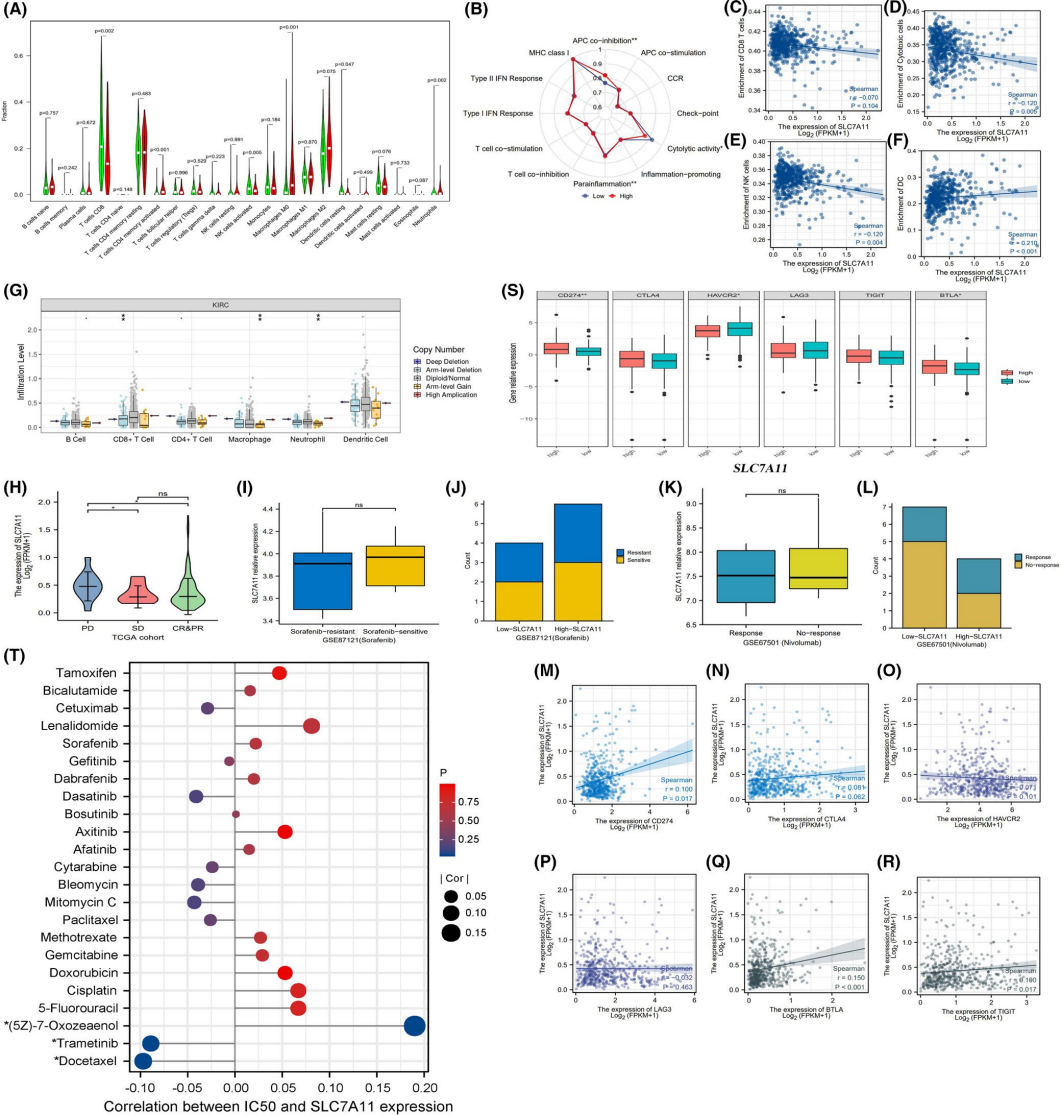
The effects of SLC7A11 on TIM and therapeutic response. (A) The differential abundances of 22 immune cells between high and low SLC7A11 expression groups. High expression group is red and low expression group is green. (B) The differences in activities of 12 immune signaling pathways between high and low expression groups. (C–F) The correlations between SLC7A11 expression and enrichments of CD8+ T cells, cytotoxic cells, NK cells, and DCs. (G) The relationships between infiltration levels of six immune cells and copy number of SLC7A11. (H) Comparison of SL7CA11 expressive levels among different clinical outcomes. (I–J) Comparison of SL7CA11 expressive levels between sorafenib‐resistant and sorafenib‐sensitive patients. (K–L) Comparison of SL7CA11 expressive levels between nivolumab response and nonresponse patients. (M–R) The expressive correlations between SLC7A11 and six immune check points. (S) The difference in expressions of six immune check points between different SLC7A11 expression levels. (T) The correlations between SLC7A11 expression and the sensitivities of multiple chemotherapy and target drugs. TIM, tumor immune microenvironment; NK, natural killer; DCs, dendritic cells; PD, progressive disease; SD stable disease; CR, complete response; PR, partial response; IC50, half maximal inhibitory concentration. *p < 0.05, **p < 0.01, and ***p < 0.001

Based on the CIBERSORT algorithm, the immune abundances of 22 leukocyte subtypes in each RCC sample are shown in Figure S2. High SLC7A11 expression resulted in decreased infiltration levels of T cells CD8, NK cells, and resting dendritic cells. Conversely, it could lead to increased enrichments of activated memory CD4 + T cells, M0 macrophages, and neutrophils (Figure 6A). Referring to previous immunology research,^26^, ^27^, ^28^, ^29^, ^30^, ^31^ immune abundance changes resulting from SLC7A11 overexpression will eventually hamper antitumor immune processes (Table 4).

**TABLE 4:** The effect of SLC7A11 on immune cell abundance

Immune cell	PMID	Basic function	Change in high expression group	Ultimate effect on antitumor immune
T cells CD8	31043744	CD8+ T cells can clear tumor cells by perforin‐granzyme and Fas/FasL pathways.	Decreased	Unfavorable
T cells CD4 memory activated	31822213	Memory T cells can maintain the cytotoxic function of CD8+ T cells, and involve in cancer surveillance.	Increased	Favorable
NK cells activated	31907401	NK cells can swiftly kill target cells by recognizing HLA class I molecules, playing a fundamental role in tumor defense.	Decreased	Unfavorable
Macrophages M0	28210073	TAMs participate in the formation of stem cell niches, immunosuppression, and cancer progression by interaction with tumor cells.	Increased	Unfavorable
Dendritic cells resting	28192720	Loss function of DCs can contribute to immune suppression in cancer.	Decreased	Unfavorable
Neutrophils	28810877 26966341	Neutrophils can recruit TAMs by releasing multiple cytokines, such as CXCR1 and CXCR2.	Increased	Unfavorable

Abbreviations: DCs, dendritic cells; NK, natural killer; TAMs, tumor‐associated macrophages.

As for immune‐related pathways, the function of antigen presentation cells and cytolytic activity were both inhibited by high SLC7A11 expression (Figure 6B). Moreover, SLC7A11 expression was negatively correlated with the enrichment of NK and cytolytic cells (Figure 6D,E), but not with that of CD8+ T cells (Figure 6C). In addition, copy number variants of SLC7A11 induced changes in the infiltration levels of CD8+ T cells, macrophages, and neutrophils (Figure 6G). These results indicate that SLC7A11 is detrimental to antitumor immunity, especially in cellular immunity.

### 3.6 SLC7A11 weakly affects therapeutic effect and sensitivities of multiple drugs

From the primary clinical outcomes of view, SLC7A11 expression in patients with PD was higher than that in patients with SD, CR, and PR (Figure 6H). Moreover, there was no significant difference in SLC7A11 expression between sorafenib‐resistant and sorafenib‐sensitive patients (Figure 6I). Although sorafenib can induce ferroptosis in cancer cells,^32^ the expression levels of SLC7A11 did not affect the treatment efficacy of sorafenib (Figure 6J).

At present, there is no definitive agreement on the predictive markers of ICI efficacy. However, it is indisputable that patients with PD‐L1 or CTLA4 overexpression can commonly benefit from ICI therapy.^33^ In view of this premise, we investigated the correlation between SLC7A11 expression and immune checkpoints. CD274 (PD‐1) (R = 0.100, *p* = 0.017), BTLA (R = 0.150, *p* < 0.001), and TIGIT (R = 0.100, *p* = 0.017) were weakly positively correlated with SLC7A11 (Figure 6M,Q,R). CTLA4, HAVCR2, and LAG3 were not correlated with SLC7A11 expression (Figure 6N–P). In addition, the expression of CD274 (PD‐L1), HAVCR2, and BTLA was clearly higher in the high SLC7A11 expression group than in the low expression group (Figure 6S). Notably, the GSE67501 cohort revealed that the expression levels of SLC7A11 did not affect the treatment efficacy of nivolumab, a PD‐1 inhibitor (Figure 6K,L).

Furthermore, based on the GSDC database, we found that SLC7A11 expression was not correlated with the sensitivity of most drugs (253/265, 95.47%) (Table S6). As shown in Figure 6T, alterations in SLC7A11 expression did not affect the sensitivity to multiple chemotherapeutic drugs, such as 5‐fluorouracil, cisplatin, gemcitabine, paclitaxel, and methotrexate. Likewise, SLC7A11 expression also had no notable impact on susceptibility to multiple target drugs, such as afatinib, sorafenib, gefitinib, dasatinib, and axitinib. Interestingly, increased expression of SLC7A11 may promote sensitivity to docetaxel and trametinib, whereas it may decrease the sensitivity to (5Z)‐7‐oxozeaenol. In summary, SLC7A11 had limited effects on the efficacy and sensitivities of multiple drugs.

### 3.7 SLC7A11 can promote the proliferation, migration, and invasion of renal cancer cells through suppressing ferroptosis

**FIGURE 7:**
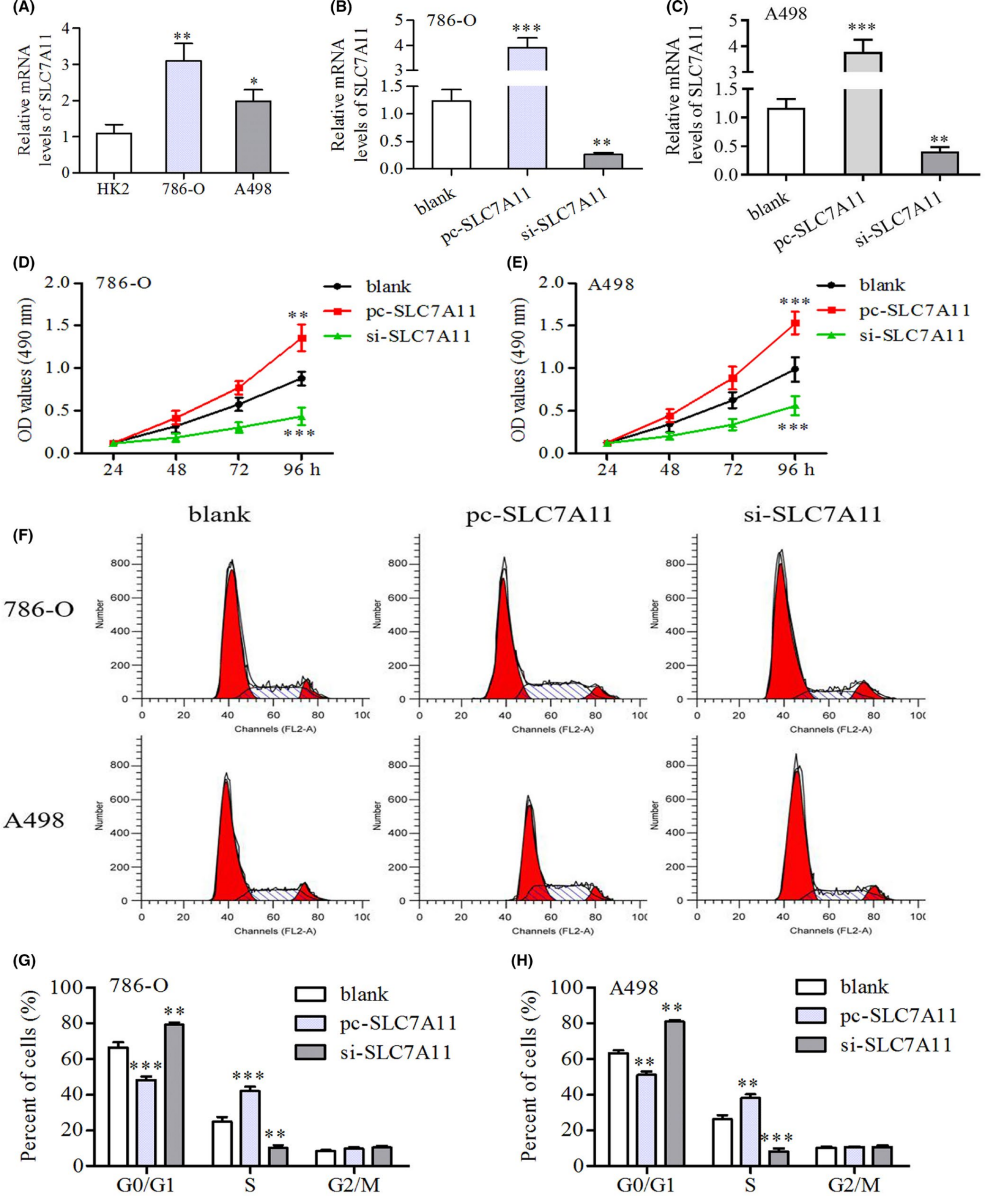
SLC7A11 can promote the proliferation of renal cancer cells. (A) Differential expressions of SLC7A11 between renal tubular epithelial cells (HK2) and renal cancer cells (786‐O and A498). (B–C) Evaluation of silencing efficiency of si‐SLC7A11. (D–E) MTT assays revealed that SLC7A11 overexpression could promote the proliferation of renal cancer cells. (F–H) Flow cytometric detection revealed that SLC7A11 overexpression could increase the S phase cells, but block the G0/G1 phase cells. *p < 0.05, **p < 0.01, and ***p < 0.001

SLC7A11 was significantly upregulated in renal cancer cells (786‐O and A498) compared to that in normal renal tubular epithelial cells (HK‐2) (Figure 7A). Specific siRNA (si‐SLC7A11) and overexpression plasmid (pc‐SLC7A11) were proven to effectively alter SLC7A11 expression (Figure 7B,C). Through MTT assays, we found that SLC7A11 overexpression promoted the proliferation of 786‐O and A498 cells, while silencing SLC7A11 had an inhibitory effect (Figure 7D–E). To go a step further, flow cytometry assays revealed the effects of SLC7A11 overexpression on cell cycle. The overexpression of SLC7A11 markedly increased the S phase population of renal cancer cells but blocked the G0/G1 phase cells (Figure 7F–H).

**FIGURE 8:**
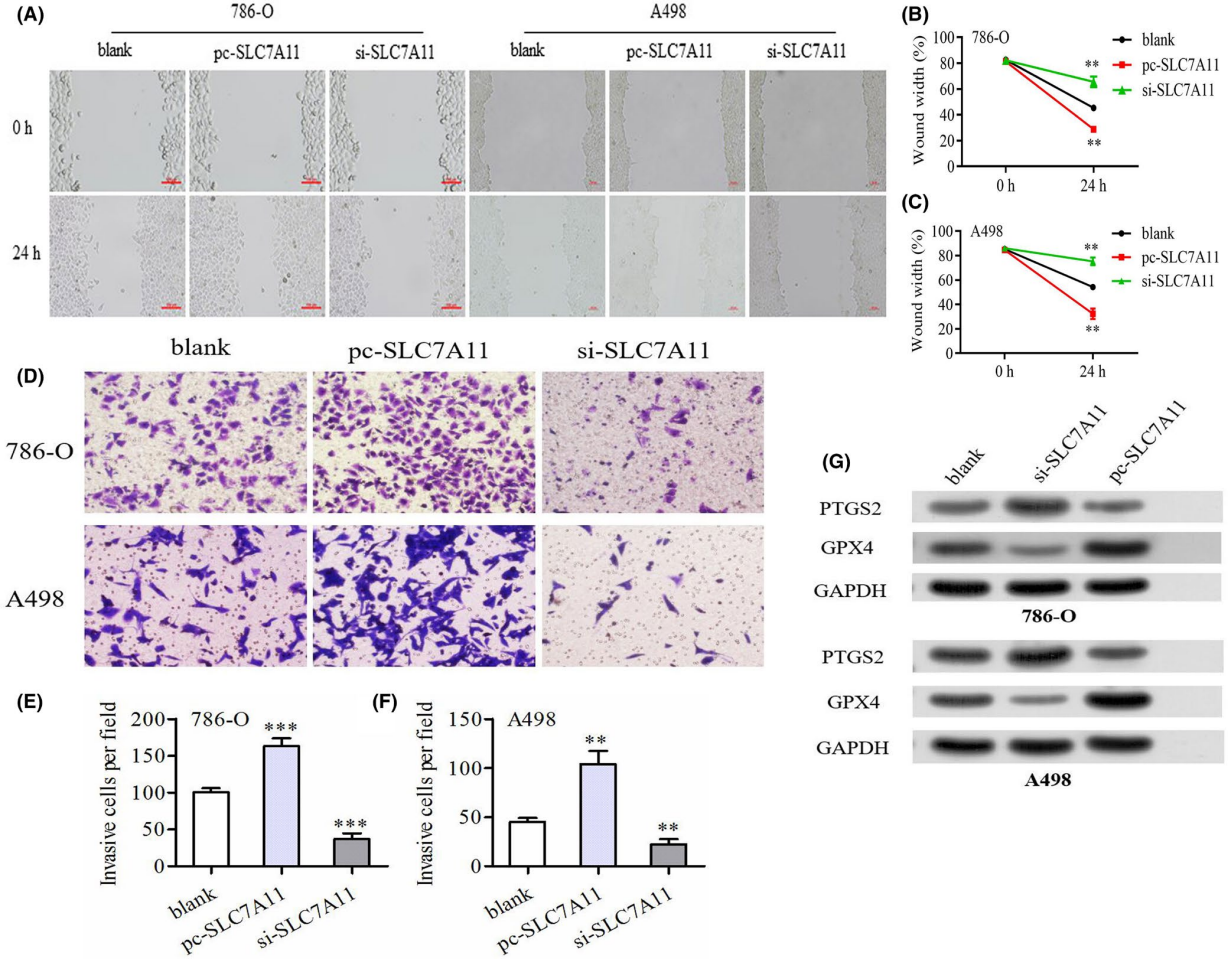
SLC7A11 has a profound influence on migration, invasion of renal cancer cells. (A–C) The wound healing assays revealed that SLC7A11 overexpression could promote the migration of renal cancer cells. (D–F) The transwell assays revealed that SLC7A11 overexpression could promote the invasion of renal cancer cells. (G) SLC7A11 overexpression could inhibit the protein expression of ferroptosis marker, PTGS2, whereas increase that of GPX4. *p < 0.05, **p < 0.01

Moreover, overexpression of SLC7A11 was enhanced, whereas silencing SLC7A11 retarded the migratory capacity of renal cancer cells (Figure 8A–C). Similarly, the invasive abilities of 789‐O and A498 cells were also enhanced by SLC7A11 overexpression and were inversely inhibited by SLC7A11 deletion (Figure 8D–F). It is well known that GPX4 and PTGS2 are often regarded as ferroptosis biomarkers in related research.^34^, ^35^ In addition, GPX4 has been proven to possess potent anti‐ferroptosis abilities owing to its antioxidant function.^36^ Western blot analysis revealed that SLC7A11 overexpression significantly increased the protein expression of GPX4 and reduced that of PTGS2, suggesting that SLC7A11 could inhibit ferroptosis by promoting GPX4 generation (Figure 8G).

## 4 DISCUSSION

Since ferroptosis was first proposed by Dixon et al. in 2013,^37^ it has great potential as an anticancer agent and has been proposed as a new treatment.^38^ Growing evidence has shown that ferroptosis regulatory genes play crucial roles in cancer progression. For example, NCOA4, a cargo receptor of iron, was found to lead to an unfavorable prognosis in RCC.^39^ SQLE, a key enzyme for lipid precursor synthesis in ferroptosis, can stimulate the malignant capabilities of pancreatic cancer cells.^40^ However, SLC7A11, as an essential link for GPX4 output, was not clearly elucidated, which was the original intention of our study.

SLC7A11 (also known as xCT), a cystine/glutamate antiporter, is capable of importing cystine for glutathione biosynthesis and antioxidant defense, thereby inhibiting ferroptosis.^9^ It is now well‐established that the well‐known tumor suppressor gene p53 can suppress tumor growth by inhibiting cystine uptake and enhancing cellular sensitivity to ferroptosis.^41^ Therefore, SLC7A11 was identified as a p53 target gene and is thought to participate in cancer progression.^42^ For example, circular RNA EPSTI1 can accelerate cervical cancer progression by sponging miR‐375 in turn targeting SLC7A11.^43^ Herein, for the first time, we confirmed that SLC7A11 could promote the proliferative, migratory, and invasive abilities of renal cancer cells. More importantly, SLC7A11 overexpression not only led to the upregulation of the ferroptosis marker, PTGS2,^34^ but also facilitated the expression of the cellular antioxidant GPX4. Interestingly, SLC7A11 was unable to regulate another vital link between ferroptosis and iron ion transport (Figure 5L). These observations confirm that SLC7A11 contributes to RCC progression by inhibiting ferroptosis. Therefore, SLC7A11 is expected to be a new target for RCC treatment.

Metabolic reprogramming is a basic characteristic of cancer. There is also a complex connection between aberrant metabolism and SLC7A11 cancer‐promoting performance. On the one hand, as mentioned above, SLC7A11 overexpression promotes cancer progression by suppressing ferroptosis. In contrast, cancer cells with SLC7A11 overexpression increased reliance on glucose and glutamine dependency, presenting potential metabolic vulnerabilities.^44^ In the present study, we found that nucleotides, fatty acids, and glycine/serine/threonine metabolisms, which provide the necessary nutrients required for cancer cell proliferation,^22^, ^23^, ^25^ were all enriched in the high SLC7A11 expression group (Figure 5). This indicated that SLC7A11 could mobilize the synthesis procedures of nucleotides, FAs, and amino acids to maintain the proliferative consumption of tumor cells. Our previous research showed that glycolysis was markedly enriched in RCC tissues compared with normal tissues because it supplies basic carbon units for DNA replication, induces therapeutic resistance in tumor cells, and maintains a high ratio of ATP/ADP for proliferation.^45^ However, glycolysis enrichment was not affected by SLC7A11 expression, which may result from metabolic plasticity caused by SLC7A11.^44^, ^46^ In summary, SLC7A11 has a complicated and profound impact on RCC metabolism.

SLC7A11 is an important supplement for RCC prognosis assessments. Currently used prognostic models cannot completely meet the demand for an accurate prediction of RCC prognosis. A key study by Westerman et al. found that commonly used 10 RCC prognostic models have limited discriminatory capacities and are inadequate for risk stratification.^47^ Therefore, given that SLC7A11 was identified as an independent prognostic factor in RCC and was able to contribute to clinical benefit (Figure 3C–E), introducing SLC7A11 into the traditional prognostic model will improve its accuracy. In addition, patients with high SLC7A11 expression were prone to recurrence and progression (Figure 6H). Hence, these patients should receive a more rigorous strategy for follow‐up and treatment, which will advance individualized management.

Interpreting the alteration of the TIM can provide important clues for exploring the mechanism of immune escape. In the current study, SLC7A11 not only led to a decline in the immune abundance of CD8+ T and NK cells, but also recruited macrophages M0 infiltrating. CD8+ T and NK cells exert potent abilities to kill tumor cells via the perforin and Fas/FasL pathways.^26^, ^28^ Macrophages can generate a supportive metastatic niche and inhibit immune‐mediated tumor clearance.^29^ In addition, M0‐like macrophages can reflect the malignancy grade of tumors, such as the malignant phenotypes of glioma.^48^ These observations support that SLC7A11 impedes antitumor cellular immunity and develops an immune‐tolerant microenvironment.

Although sorafenib can exert anticancer effects by inhibiting ferroptosis^49^ and hindering ferroptosis can facilitate sorafenib resistance,^50^ we found that SLC7A11 failed to correlate with the curative effect and sensitivity of sorafenib (Figure 6I,J,T). The possible reason is that sorafenib exerts antitumor effects by directly targeting xCT activity^51^; therefore, it may not be an appropriate drug for therapeutic response prediction. Moreover, SLC7A11 was weakly associated with immune checkpoints, and its expression did not affect the nivolumab response (Figure 6K,L). Therefore, SLC7A11 may not serve as a biomarker for predicting the efficacy of sorafenib and ICIs. As for chemotherapy and target drugs, SLC7A11 could not affect the sensitivities of multiple classic drugs, such as 5‐fluorouracil, cisplatin, paclitaxel, gemcitabine, gefitinib, and sorafenib. Overall, SLC7A11 has limited effects on the efficacy and sensitivity of multiple drugs.

Naturally, there are some limitations to this study. First, the prognostic value of SLC7A11 should be validated in a clinical cohort. Second, the mRNA and protein expression levels of SLC7A11 were not measured in the clinical specimens. Third, we did not analyze the oncogenic mechanism of SLC7A11 in depth. Fourth, the cancer‐promoting feature of SLC7A11 was not evaluated in vitro using a xenograft model.

## 5 CONCLUSIONS

In conclusion, we comprehensively investigated the functions of SLC7A11 in prognosis, immune microenvironment, biological metabolism, therapeutic correlation, and pro‐oncogenic abilities of RCC. SLC7A11 is markedly upregulated in multiple cell subtypes of renal cancer and is an unfavorable prognostic factor for RCC. SLC7A11 not only contributed to RCC prognostic assessment, but also resulted in metabolic reprogramming to fulfill the biosynthetic demands of tumor proliferation. Although high SLC7A11 expression retarded antitumor immunity, it was not related to the efficacy of sorafenib and ICIs. More importantly, we confirmed that SLC7A11 could promote the proliferation, migration, and invasion of renal cancer cells by enhancing GPX4 output, which in turn inhibits ferroptosis. Altogether, our findings reveal the comprehensive and complex oncogenic abilities of SLC7A11 in RCC, which provides important clues for the clinical assessment and molecular mechanism of RCC.

## CONFLICT OF INTERESTS

The authors declare that they have no competing interests.

## ETHICAL DISCLOSURE

Not applicable.

## CONSENT FOR PUBLICATION

Not applicable.

## Supporting information

Fig S1

Fig S2

Table S1

Table S2

Table S3

Table S4

Table S5

Table S6

## Data Availability

The datasets used and/or analyzed in the current study are available from the corresponding author upon reasonable request.
