# Supplementary figures and images for: The roles of ferroptosis regulatory gene SLC7A11 in renal cell carcinoma: A multi‐omics study
Source: Cancer Med. 2021 Nov 10;10(24):9078–96. doi: 10.1002/cam4.4395 (PMC8683539; doi:10.1002/cam4.4395)

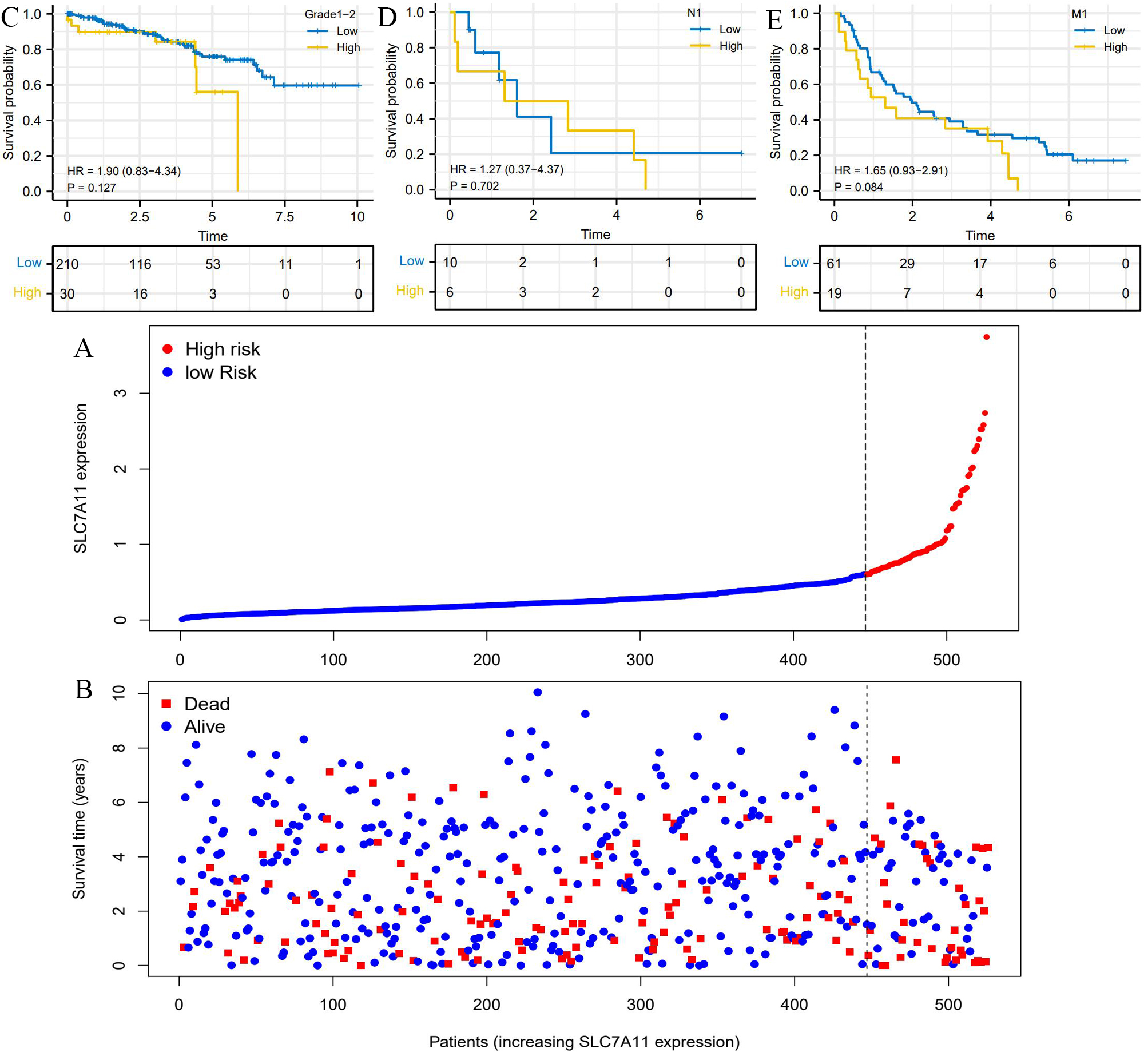

Supplement: Supplementary file 1 — Fig S1 [file CAM4-10-9078-s008.jpg]

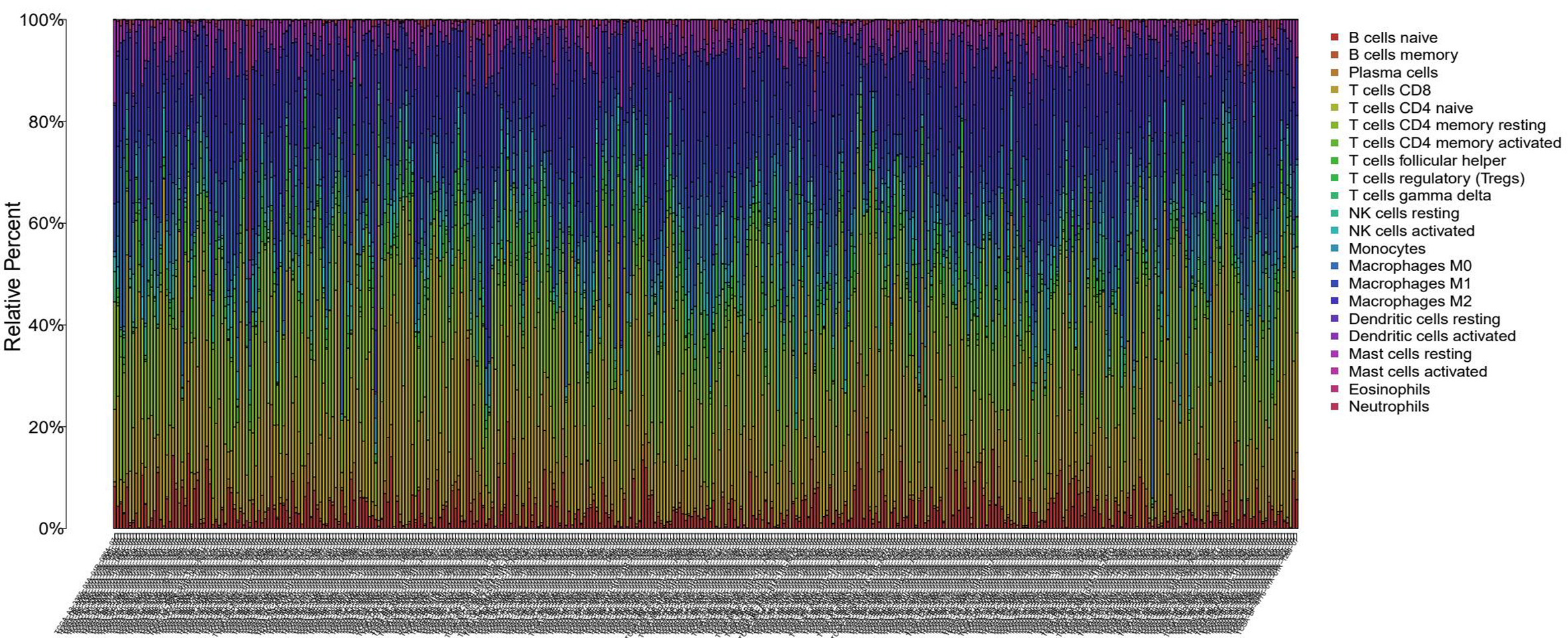

Supplement: Supplementary file 2 — Fig S2 [file CAM4-10-9078-s002.jpg]
