# Supplementary material for: The roles of ferroptosis regulatory gene SLC7A11 in renal cell carcinoma: A multi‐omics study
Source: Cancer Med. 2021 Nov 10;10(24):9078–96. doi: 10.1002/cam4.4395 (PMC8683539; doi:10.1002/cam4.4395)
Supplement: Supplementary file 3 — Table S1 [file CAM4-10-9078-s004.docx]

Supplementary Table 1. Description of the gene sets for GSEA

| Names | Gene counts | Description |
| --- | --- | --- |
| ***Glycolysis metabolism*** |  |  |
| Hallmark Glycolysis | 200 | Genes encoding proteins involved in glycolysis and gluconeogenesis |
| Reactome Glycolysis | 72 | Glycolysis |
| GO Glycolytic Process | 106 | Glycolysis begins with the metabolism of a carbohydrate to generate products that can enter the pathway and ends with the production of pyruvate |
| Module 306 | 26 | Glycolysis and TCA cycle. |
| ***Biosynthetic metabolisms*** |  |  |
| Hallmark Fatty acid Metabolism | 158 | Genes encoding proteins involved in metabolism of fatty acids |
| KEGG Glycine Serine and Threonine Metabolism | 31 | Glycine, serine and threonine metabolism |
| Reactome Glutamate and Glutamine Metabolism | 14 | Glutamate and glutamine metabolism |
| Module 337 | 64 | Nucleotide metabolism |
| ***Ferroptosis process*** |  |  |
| WP- Ferroptosis | 40 | Ferroptosis |
| Hallmark Oxidative Phosphorylation | 200 | Genes encoding proteins involved in oxidative phosphorylation |
| Hallmark Reactive Oxygen Species Pathway | 49 | Genes up-regulated by reactive oxygen species (ROS) |
| GOBP Iron ion Transport | 79 | The directed movement of iron (Fe) ions into, out of or within a cell, or between cells, by means of some agent such as a transporter or pore |

GSEA, gene set enrichment analysis; GO, Gene Ontology.
