# Supplementary material for: The roles of ferroptosis regulatory gene SLC7A11 in renal cell carcinoma: A multi‐omics study
Source: Cancer Med. 2021 Nov 10;10(24):9078–96. doi: 10.1002/cam4.4395 (PMC8683539; doi:10.1002/cam4.4395)
Supplement: Supplementary file 4 — Table S2 [file CAM4-10-9078-s003.docx]

Supplementary Table 2. The primer lists

| Gene | Primer | Sequence (5' -> 3') |
| --- | --- | --- |
| SLC7A11 | Forward | 5'‐CAGTAGCTGCAGGGCGTA‐3 |
|  | Reverse | 5'‐ACCCGCTGTTGTACGAGTC‐3' |
| GAPDH | Forward | 5'‐TGCACCACCAACTGCTTAGC‐3 |
|  | Reverse | 5'‐GGCATGGACTGTGGTCATGA‐3' |
