# Supplementary material for: The roles of ferroptosis regulatory gene SLC7A11 in renal cell carcinoma: A multi‐omics study
Source: Cancer Med. 2021 Nov 10;10(24):9078–96. doi: 10.1002/cam4.4395 (PMC8683539; doi:10.1002/cam4.4395)
Supplement: Supplementary file 5 — Table S3 [file CAM4-10-9078-s007.docx]

Supplementary Table 3. Clinical characteristics of 537 RCC patients in TCGA cohort

| Variables | Number (percentage) |
| --- | --- |
| Vital status |  |
| Alive | 337 (62.8%) |
| Dead | 200 (37.2%) |
| Age |  |
| ＜60 | 247 (46.0%) |
| ≥60 | 290 (54.0%) |
| Gender |  |
| Male | 346 (64.4%) |
| Female | 191 (35.6%) |
| Tumor Grade |  |
| G1 | 14 (2.6%) |
| G2 | 230 (42.8%) |
| G3 | 207 (38.5%) |
| G4 | 78 (14.5%) |
| Gx | 5 (1.0%) |
| Unknow | 3 (0.6%) |
| Clinical Stage |  |
| Stage I | 269 (50.1%) |
| Stage II | 57 (10.6%) |
| Stage III | 125 (23.3%) |
| Stage IV | 83 (15.4%) |
| Unknow | 3 (0.6%) |
| T stage |  |
| T1 | 275 (51.2%) |
| T2 | 69 (12.8%) |
| T3 | 182 (33.9%) |
| T4 | 11 (2.1%) |
| M stage |  |
| M0 | 426 (79.3%) |
| M1 | 79 (14.7%) |
| Mx | 30 (5.6%) |
| Unknow | 2 (0.4%) |
| N stage |  |
| N0 | 240 (44.7%) |
| N1 | 17 (3.2%) |
| Nx | 280 (52.1%) |

RCC, renal cell carcinoma; TCGA, The Cancer Genome Atlas.
