# Supplementary material for: The roles of ferroptosis regulatory gene SLC7A11 in renal cell carcinoma: A multi‐omics study
Source: Cancer Med. 2021 Nov 10;10(24):9078–96. doi: 10.1002/cam4.4395 (PMC8683539; doi:10.1002/cam4.4395)
Supplement: Supplementary file 6 — Table S4 [file CAM4-10-9078-s001.docx]

Supplementary Table 4. Clinical characteristics of 91 RCC patients in ICGC cohort

| Variables | Number (percentage) |
| --- | --- |
| Vital status |  |
| Alive | 61 (67.0%) |
| Dead | 30 (33.0%) |
| Age |  |
| ＜60 | 39 (42.9%) |
| ≥60 | 52 (57.1%) |
| Gender |  |
| Male | 52 (57.1%) |
| Female | 39 (42.9%) |
| Tumor Grade | Unknow |
| Clinical Stage |  |
| Stage I | 48 (52.7%) |
| Stage II | 12 (13.1%) |
| Stage III | 15 (16.5%) |
| Stage IV | 9 (9.9%) |
| Unknow | 7 (7.8%) |
| T stage |  |
| T1 | 54 (59.3%) |
| T2 | 13 (14.3%) |
| T3 | 22(24.2%) |
| T4 | 2 (2.2%) |
| M stage |  |
| M0 | 81 (89.0%) |
| M1 | 9 (9.9%) |
| Mx | 1 (1.1%) |
| N stage |  |
| N0 | 80(87.9%) |
| N1 | 2 (2.2%) |
| Nx | 9 (9.9%) |

RCC, renal cell carcinoma; ICGC, International Cancer Genome Consortium.
