# Supplementary material for: The roles of ferroptosis regulatory gene SLC7A11 in renal cell carcinoma: A multi‐omics study
Source: Cancer Med. 2021 Nov 10;10(24):9078–96. doi: 10.1002/cam4.4395 (PMC8683539; doi:10.1002/cam4.4395)
Supplement: Supplementary file 7 — Table S5 [file CAM4-10-9078-s006.docx]

Supplementary Table 5. Clinical characteristics of 39 RCC patients in GSE29609 cohort

| Variables | Number (percentage) |
| --- | --- |
| Vital status |  |
| Alive | 16 (41.0%) |
| Dead | 23 (59.0%) |
| Age |  |
| ＜60 | 15 (38.5%) |
| ≥60 | 24 (61.5%) |
| Gender | Unknow |
| Tumor Grade |  |
| G1 | 1 (2.5%) |
| G2 | 12 (30.8%) |
| G3 | 11 (28.2%) |
| G4 | 15 (38.5%) |
| T stage |  |
| T1 | 11 (28.2%) |
| T2 | 5 (12.9%) |
| T3 | 22 (56.4%) |
| T4 | 1 (2.5%) |
| M stage |  |
| M0 | 25 (64.1%) |
| M1 | 14 (35.9%) |
| N stage |  |
| N0 | 31 (79.5%) |
| N1 | 8 (20.5%) |

RCC, renal cell carcinoma.
